# Effects of anti-osteoporotic drugs in patients with chronic kidney disease: a systemic review and network meta-analysis of bone mineral density, clinical fracture rate and renal function

**DOI:** 10.3389/fphar.2025.1569744

**Published:** 2025-06-17

**Authors:** Chih-Hsuan Wung, Hao-Yu Liu, Tsung-Ting Tsai, Jia-Jin Chen

**Affiliations:** ^1^ Department of Orthopedic Surgery, Kaohsiung Chang Gung Memorial Hospital, Kaohsiung, Taiwan; ^2^ Department of Orthopedic Surgery, Linkuo Chang Gung Memorial Hospital, Taoyuan, Taiwan; ^3^ Department of Orthopaedic Surgery, Spine Section and Bone and Joint Research Center, Chang Gung Memorial Hospital, Taoyuan, Taiwan; ^4^ School of Medicine, Chang Gung University, Taoyuan, Taiwan; ^5^ Kidney Research Center, Nephrology Department, Linkou Chang Gung Memorial Hospital, Taoyuan, Taiwan

**Keywords:** osteoporosis, chronic kidney disease, anti-osteoporotic drugs, bone mineral density, fracture, renal function

## Abstract

**Objective:**

This systematic review and network meta-analysis investigated the effects of various anti-osteoporotic drugs (AODs) on bone mineral density (BMD), estimated glomerular filtration rate (eGFR), and clinical fracture rate in patients with chronic kidney disease (CKD) and osteoporosis.

**Methods and results:**

We searched for relevant studies in PubMed, Embase, and Web of Science and included randomized controlled trials with any following outcomes of interest: clinical fracture rate, BMD, and eGFR. The effectiveness of different AODs was assessed by random-effects model network meta-analysis and ranked on the basis of P-scores. A total of seven studies involving 18,503 patients were included. Three AODs: sclerostin inhibitors, bisphosphonates and parathyroid hormone (PTH) analogs were associated with mild but significantly increased BMD at the lumbar spine, total hip, and femoral neck. In addition, sclerostin inhibitors (relative risk; RR:0.38, 95% CI: 0.23–0.62), bisphosphonates (RR:0.53, 95% CI: 0.30–0.92), denosumab (RR:0.58, 95% CI: 0.52–0.66), and PTH analogs (RR:0.68, 95% CI: 0.55–0.86) effectively reduced clinical fracture rates. AODs did not significantly affect eGFRs. Among the five AODs, according to P-score ranking, sclerostin inhibitors were the most effective in reducing clinical fracture risk, and PTH analogs resulted in the most favorable improvement in BMD. The five AODs had no significant effect on eGFR.

**Conclusion:**

We demonstrated that bisphosphonates, PTH analogs, denosumab, and sclerostin inhibitors can reduce clinical fracture risk in CKD patient’s osteoporosis but with low to very low confidence of evidence. In clinical practice, sclerostin inhibitors and PTH analogs could result in the highest reduction in clinical fracture risk and improvement in BMD, respectively.

## 1 Introduction

Osteoporosis is a global health concern that is characterized by a slow and silent progression (Rachner et al., 2011; Sheik Ali, 2023). The main feature of osteoporosis is bone fragility, which is caused by bone mass loss and a deterioration of the bone microarchitecture (Wung et al., 2021). Patients with chronic kidney disease (CKD) often experience mineral and bone disorders, which include abnormalities in serum calcium, phosphorus, and parathyroid hormone (PTH) levels and vitamin D metabolism as well as disturbances in bone turnover and mineralization (Ginsberg and Ix, 2022). As CKD progresses from stage 1–5, the incidence of fractures increases from 15.0 to 20.5, 24.2, 31.2, and 46.3/1000 person-years, respectively (Pimentel et al., 2021). The impaired bone quality in patients with CKD increases their risk of osteopenia, osteoporosis, and bone fractures (Moe, 2017). Both nonvertebral and vertebral fractures are strongly associated with reductions in bone mineral density (BMD) at various sites, such as the femoral neck, total hip, and lumbar spine (Compston et al., 2019). The clinical diagnosis of osteoporosis is primarily based on BMD measurements through dual-energy X-ray absorptiometry (Elonheimo et al., 2021). Variations in BMD at different sites allow nephrologists and orthopedic surgeons to tailor antiosteoporosis strategies for reducing osteoporosis-related morbidity and mortality (Mohammed and Loay, 2023).

Currently, anti-osteoporotic drugs (AODs) used in pharmacological treatments are classified as antiresorptive agents, anabolic agents, and strontium ranelate (Hara et al., 2021). Antiresorptive agents include bisphosphonates, receptor activator of nuclear factor-kappa B ligand (RANKL) inhibitors, and selective estrogen receptor modulators (SERMs). Anabolic agents include PTH analogs and sclerostin inhibitors (Sindel, 2023; Wung et al., 2023). Bisphosphonates, commonly used as a first-line treatment, reduce the risk of fractures by targeting osteoclast function (Kuznik et al., 2020). Denosumab, a humanized monoclonal antibody, is a RANKL inhibitor that reduces osteoclast function by preventing RANKL–osteoclast binding (Zhang et al., 2020). In addition, denosumab is mainly used in postmenopausal women with osteoporosis (Deeks, 2018). SERMs, such as raloxifene and bazedoxifene, can also prevent bone loss in postmenopausal women with osteoporosis (LeBoff et al., 2022). PTH analogs, including teriparatide and abaloparatide, act as PTH receptor agonists, enhancing bone remodeling and stimulating bone formation (Winzenrieth et al., 2021). Romosozumab, another humanized monoclonal antibody, serves as a sclerostin inhibitor with the dual effect of increasing bone formation and reducing bone resorption (Bandeira and Lewiecki, 2022). Strontium ranelate exerts a dual effect by inducing bone formation and inhibiting bone resorption (Kołodziejska et al., 2021).

Numerous studies have explored the efficacy and safety of AODs, including bisphosphonates (Haarhaus et al., 2023), denosumab (Broadwell et al., 2021), and PTH analogs (Bilezikian et al., 2019; Haarhaus et al., 2023; Schroeder et al., 2023), for treating osteoporosis in the general population, postmenopausal women, and patients with different CKD stages. Although several AODs are available for clinical use, few studies have compared the effects of AODs on BMD outcomes and fracture risk reduction in patients with CKD. In addition, the effect of AODs on CKD progression remains unclear and most direct comparison between AODs in CKD population is lack. Thus, we conducted a comprehensive systematic review and network meta-analysis (NMA) to investigate the effects of different AODs on BMD, fracture risk reduction, and estimated glomerular filtration rate (eGFR) preservation in patients with CKD.

## 2 Materials and methods

### 2.1 The literature search strategy

We performed this NMA in accordance with the Preferred Reporting Items for Systematic Reviews and Meta-Analyses (PRISMA) (Page et al., 2021) Extension for NMAs guidelines (Supplementary Table S1). The study design and protocol were registered in PROSPERO (CRD42023476047).

Two authors (C-H W and H-Y L) independently screened for studies published in PubMed, Embase, and Web of Science before June 2023 using same protocol and results (Supplementary Table S2). Our search strategy focused on randomized clinical trials investigating the effects of different interventions on BMD improvement, adverse event rates, and eGFR preservation in patients with CKD. Supplementary Table S2 presents the search strategy and process in detail. Although we excluded review articles and meta-analyses from our analysis, their references were examined to identify relevant studies. Any disagreements were resolved and search strategies were reviewed by a third reviewer (J-J C). There is no article type or language limitation.

### 2.2 Study eligibility criteria

Two authors (C-H W and H-Y L) independently conducted a search and reviewed the titles and abstracts of identified studies. We initially excluded articles whose titles or abstracts were irrelevant to the objective of the current study. Furthermore, we assessed the eligibility of studies by examining their full texts. We included studies if they involved adult patients with nondialysis CKD stages 1–5 and severe osteopenia or osteoporosis based on World Health Organization guidelines (osteoporosis: T score below −2.5 standard deviations; osteopenia: T score between −1 and −2.5 standard deviations) (Sozen et al., 2017) and compared outcomes between groups of patients receiving different types of AODs or a placebo. The primary intervention evaluated was the administration of AODs, namely (1) bisphosphonates (clodronate, tiludronate, alendronate, risedronate, ibandronate, pamidronate, zoledronate, and etidronate); (2) RANKL inhibitors (denosumab); (3) SERMs (raloxifene and bazedoxifene); (4) PTH analogs (teriparatide and abaloparatide); (5) sclerostin inhibitors (romosozumab); and (6) strontium ranelate. We did not impose any search restrictions on drug dosage or article language. A third reviewer (J-J C) was responsible for resolving any disagreement regarding the eligibility of studies.

### 2.3 Data extraction and outcomes

Two authors (C-H W and H-Y L) extracted data by using a standardized data abstraction form. The following data were extracted: country of origin, year of publication, study characteristics, patient demographics, sample size, and follow-up duration. In addition, data on changes in clinical fracture rates (primary outcome), eGFR, and BMD at the femoral neck, total hip, and lumbar spine were extracted. To analyze changes in BMD, we prioritized data reflecting 1-year BMD changes after intervention. In cases where 1-year BMD data were unavailable, we extracted and used data on BMD changes at 6 months after the intervention.

### 2.4 Data synthesis and statistical analysis

The characteristics of the enrolled patients, interventions, and outcomes of interest were extracted from the included studies (Table 1). To evaluate the preventive effect of different AODs on clinical fractures, we calculated risk ratios (RRs) for pooled outcomes. To evaluate the effect of AODs on BMD and eGFR, we calculated the mean difference. We conducted this frequentist NMA with a random-effects model by using the netmeta package in R version 4.0.2 (R Core Team, Vienna, Austria). Heterogeneity was examined using the I2 statistic and the chi-square test. Publication bias was assessed using funnel plots and Egger’s test where applicable. The results of the NMA and direct comparisons were summarized in forest plots and league tables. We used the P-score method to determine the probability of an AOD being more effective than others. Incoherence was evaluated using a design-by-treatment interaction model, with p values greater than 0.1 indicating no concern regarding incoherence (Higgins et al., 2012; Nikolakopoulou et al., 2020). Risk of bias in the included studies was assessed using the RoB 2 tool (Sterne et al., 2019). Confidence in the evidence of this NMA regarding the effectiveness of AODs in preventing clinical fractures was evaluated using the confidence in NMA framework (Nikolakopoulou et al., 2020). Analyses were conducted in R, version 4.2.2 (R Program for Statistical Computing [31 October 2022]), with netmeta package.

**TABLE 1:** Characteristics of enrolled studies.

Name, year	Cummings, 2009	Haghverdi, 2013	Kenneth, 2017	Langdaul, 2022	Miller, 2007	Miller, 2016	Toussaint, 2010
Country	United States	Iran	Multi-countries	Multi-countries	United States	United States	Australia
Design	Double-blind	Not blind	Double-blind	Double-blind	Double-blind	Double-blind	Double-blind
Arm	2	2	2	2	2	3	2
Female (%)	100	100	100	100	100	100	34
Mean Age	72.3	62.8	74.3	70.4	69.1	68.8	62.6
N	7808	60	4093	3013	1637	2463	50
Inclusion criteria	1. 60∼90-year-old women 2. BMD T score −2.5∼-4.0	1. Longer than 1 year of menopause 2. Age >40 years 3. Osteoporosis or severe osteopenia	Ambulatory postmenopausal women 55–90 years of age and met one of following criteria:1. BMD T score < -2.5 + 1 moderate to severe vertebral fracture 2. BMD T score < -2.5 + 2 mild vertebral fracture 3. BMD T score < -2.0 + 2 moderate to severe vertebral fracture 4. BMD T score < -2.0 + fracture of proximal femur sustained 3–24 months before randomization	1. 55∼90-year-old women 2. BMD T score −2.5∼-3.5	1. Ambulatory postmenopausal women 2. Serum creatinine <2 mg/dL	1. Postmenopausal women aged 49-86 2. Osteoporosis according to WHO criteria	1. Age 18–80 years 2. eGFR: 20∼60 3. Creatinine clearance: 25 mL/min (CKD stage 3–4)
Intervention-1	Denosumab (RANK ligand)	Raloxifene	Romosozumab	Romosozumab	Teriparatide	Abaloparatide	Alendronate
Intervention-2	N/A	N/A	Alendronate	N/A	N/A	Teriparatide	N/A
Control	Placebo	Placebo	—	Placebo	Placebo	Placebo	Placebo
Follow-up time (month)	36	8	24	12	18	18	18

Abbreviations: BMD, bone mineral density; CKD, chronic kidney disease; eGFR, estimated glomerular filtration rate; N, number; N/A, not applicable; WHO, world health organization.

## 3 Results

### 3.1 Results of the search

**FIGURE 1:**
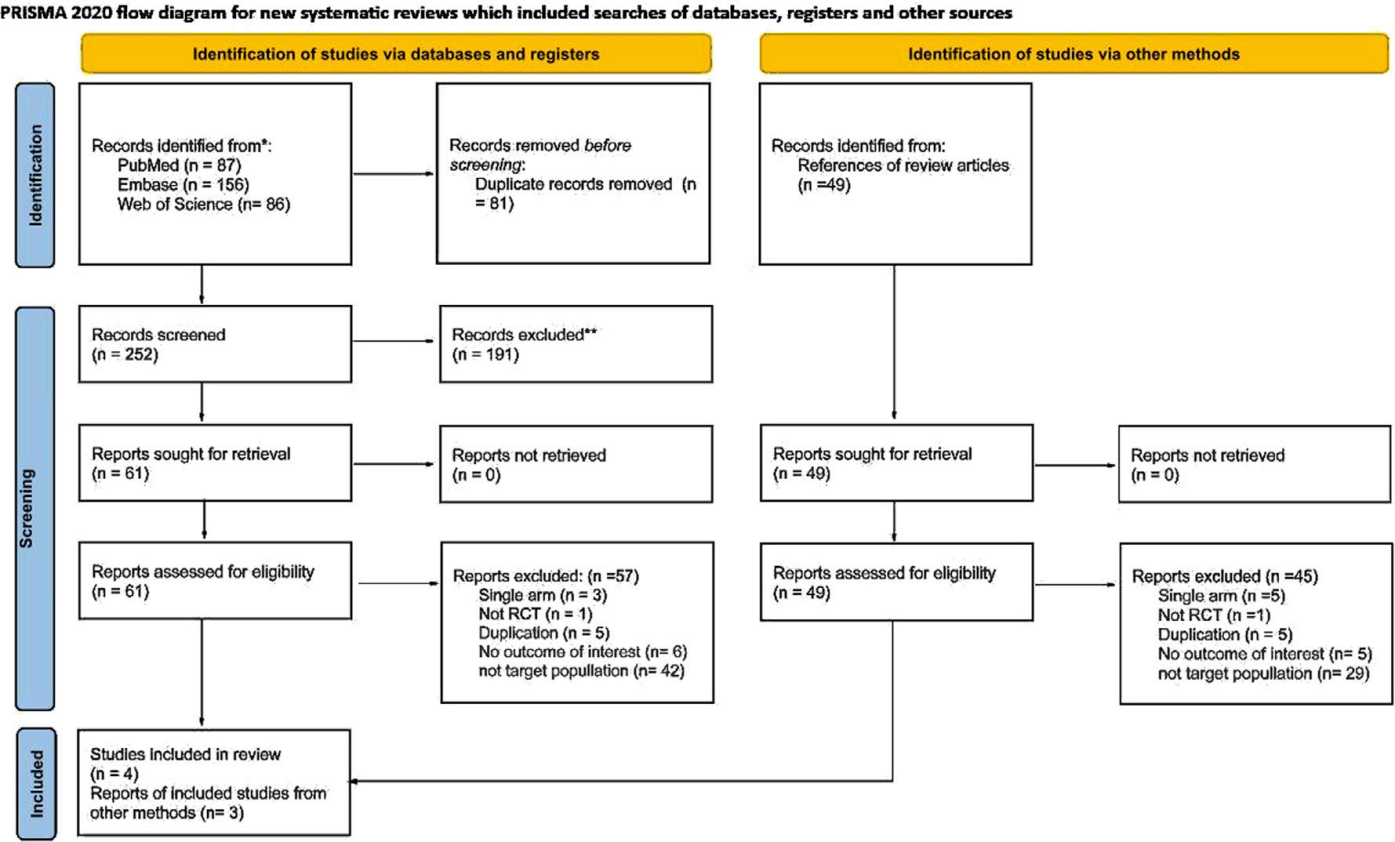
PRISMA flow chart.

We identified relevant studies from PubMed, Embase, and Web of Science. After the exclusion of duplicate articles, the remaining 252 articles were subjected to a preliminary screening based on their titles and abstracts. This process resulted in 61 relevant articles. Subsequently, we conducted a thorough full-text review and finally included four articles in our analysis. Furthermore, a comprehensive search of references in the included articles yielded an additional three publications. Figure 1 presents a detailed overview of the literature search process and the PRISMA flowchart. Supplementary Table S2 details the search strategy for each database. Supplementary Table S4 provides details of the excluded studies.

### 3.2 Study characteristics

This NMA included seven randomized controlled trials involving a total of 18,503 patients. The sample sizes ranged from 25 to 3,906 individuals. Five studies were conducted in a single country (Cummings et al., 2009; Haghverdi et al., 2014; Miller et al., 2016; Miller et al., 2007; Toussaint et al., 2010), whereas two were multinational studies (Langdahl et al., 2022; Saag et al., 2017). The mean age of the patients was 71.7 years, and the follow-up duration varied from 6 to 36 months. Of the seven studies, six primarily included postmenopausal women (Cummings et al., 2009; Haghverdi et al., 2014; Langdahl et al., 2022; Miller et al., 2016; Miller et al., 2007; Saag et al., 2017), accounting for 18,410 female patients or 99.5% of the total study population. Only one study included male patients (Toussaint et al., 2010). The average eGFR of the patients was 73.9 ± 19.1 mL/min. Most of the studies involved patients with CKD stages 1–4 (Cummings et al., 2009; Langdahl et al., 2022; Miller et al., 2016; Miller et al., 2007; Saag et al., 2017; Toussaint et al., 2010), whereas one article primarily included patients with CKD stage 5 (Haghverdi et al., 2014). In addition, six studies included patients with severe osteopenia or osteoporosis. One study initially had three arms; however, because we combined drugs with the same mechanisms into one group, we analyzed it as a two-arm study (Miller et al., 2016). Table 1 lists the characteristics of the seven included studies.

### 3.3 Clinical fractures

**FIGURE 2:**
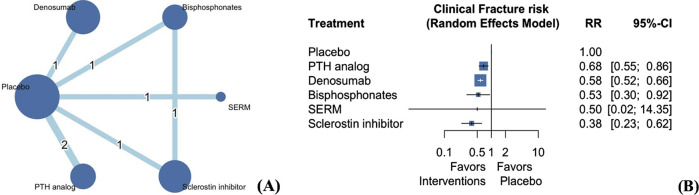
Network plot **(A)** and forest plot **(B)** of eligible comparisons among AODs for clinical fractures. Footnote: The network plot presents each intervention as a node, with connecting lines indicating direct comparisons between different AODs. The size of each node reflects the total number of participants receiving that intervention, while the thickness of the connecting lines corresponds to the number of studies enrolled to each direct comparison. The numerical label on each line denotes the number of studies included in that specific comparison. Abbreviations: AOD, anti-osteoporotic drugs; PTH, parathyroid hormone; SERM, selective estrogen receptor modulator; MD, mean difference; RR, risk ratio; CI, confidence interval.

The seven included studies investigated the risk of clinical fractures of the five types of AODs (Figure 2A) (Cummings et al., 2009; Haghverdi et al., 2014; Langdahl et al., 2022; Miller et al., 2016; Miller et al., 2007; Saag et al., 2017; Toussaint et al., 2010). The results revealed that the five AODs significantly reduced the risk of clinical fractures when compared with placebo (Figure 2B; Supplementary Table S4; PTH analogs: RR 0.68, 95% CI 0.55 to 0.86; denosumab: RR 0.58, 95% CI 0.52 to 0.66; bisphosphonates: RR 0.53, 95% CI 0.30 to 0.92; SERMs: RR 0.50, 95% CI 0.02 to 14.35; sclerostin inhibitor: RR 0.38, 95% CI 0.23–0.62). Treatment efficacy was ranked on the basis of P-scores. Sclerostin inhibitors exhibited the highest treatment efficacy, followed by bisphosphonates, denosumab, SERM, PTH analog, and placebo (Supplementary Table S5). The quantification of heterogeneity yielded an I2 value of 0% (0.0%–89.6%; P = 0.77).

### 3.4 Change in BMD from baseline

#### 3.4.1 Lumbar spine

**FIGURE 3:**
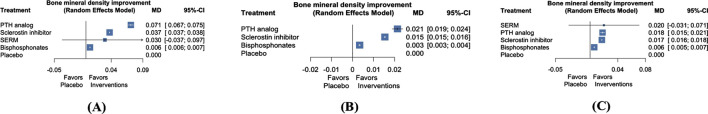
Forest plots of NMA for the comparison of AODs on BMD changes at the lumbar spine **(A)**, total hip **(B)**, and femoral neck **(C)**. Abbreviations: NMA, network meta-analysis; BMD, bone mineral density; PTH, parathyroid hormone; SERM, selective estrogen receptor modulator; MD, mean difference; CI, confidence interval.

Four studies involving 9,629 patients examined the change in BMD at the lumbar spine (Haghverdi et al., 2014; Langdahl et al., 2022; Miller et al., 2016; Saag et al., 2017) following treatment with four types of AODs (Supplementary Figure S1). Our analysis revealed that the three AODs significantly improved BMD at the lumbar spine compared with placebo (Figure 3A; Supplementary Table S6; PTH analog: mean difference 0.071, 95% CI 0.067 to 0.075; sclerostin inhibitor: mean difference 0.037, 95% CI 0.037 to 0.038; bisphosphonates: mean difference 0.006, 95% CI 0.006–0.007). Treatment efficacy was ranked on the basis of P-scores. PTH analogs exhibited the highest treatment efficacy, followed by sclerostin inhibitors, SERMs, bisphosphonates, and placebo (Supplementary Table S5).

#### 3.4.2 Total hip

Three articles involving 9,569 patients examined the effect of three types of AODs on the change in BMD at the total hip (Supplementary Figure S1) (Langdahl et al., 2022; Miller et al., 2016; Saag et al., 2017). Our analysis revealed that the three AODs significantly improved BMD at the total hip when compared with placebo (Figure 3B; Supplementary Table S7; PTH analog: mean difference 0.021, 95% CI 0.019 to 0.024; sclerostin inhibitors: mean difference 0.015, 95% CI 0.015 to 0.016; bisphosphonates: mean difference 0.003, 95% CI 0.003–0.004). Treatment efficacy was ranked on the basis of P-scores. PTH analogs exhibited the highest treatment efficacy, followed by sclerostin inhibitors, bisphosphonates, and placebo (Supplementary Table S5).

#### 3.4.3 Femoral neck

Four articles involving 9,629 patients investigated the effect of four AODs on the change in BMD at the femoral neck (Supplementary Figure S3) (Haghverdi et al., 2014; Langdahl et al., 2022; Miller et al., 2016; Saag et al., 2017). Our analysis revealed that the three AODs significantly improved BMD at the femoral neck compared with placebo (Figure 3C; Supplementary Table S8; PTH analogs: mean difference 0.018, 95% CI 0.015 to 0.021; sclerostin inhibitors: mean difference 0.017, 95% CI 0.016 to 0.018; bisphosphonates: mean difference 0.006, 95% CI 0.005–0.007). Treatment efficacy was evaluated on the basis of P-scores. PTH analogs exhibited the highest effectiveness, followed by sclerostin inhibitors, SERMs, bisphosphonates, and placebo (Supplementary Table S5).

Permission must be obtained for use of copyrighted material from other sources (including the web). Please note that it is compulsory to follow figure instructions.

### 3.5 eGFR

**FIGURE 4:**
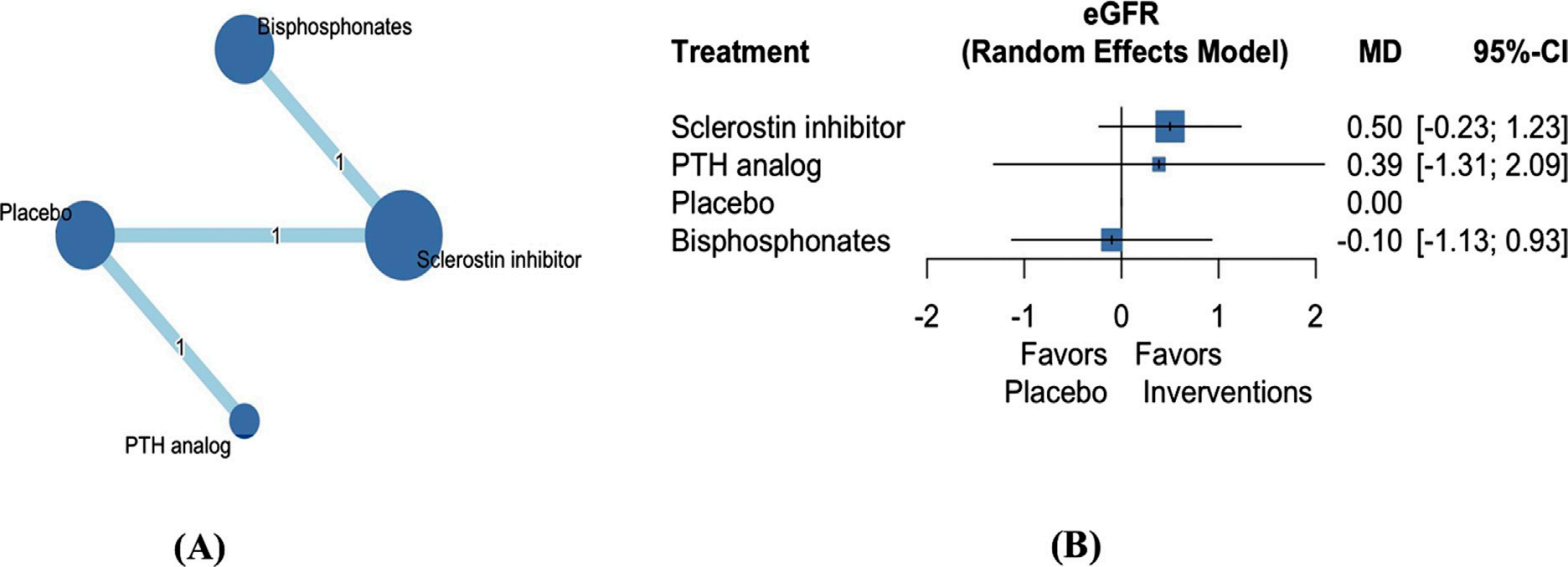
Network plot **(A)** and forest plot **(B)** of eligible comparisons among AODs for eGFR change. Abbreviations: AOD, anti-osteoporotic drugs; eGFR, estimated glomerular filtration rate; PTH, parathyroid hormone; MD, mean difference; CI, confidence interval. Footnote: The network plot presents each intervention as a node, with connecting lines indicating direct comparisons between different AODs. The size of each node reflects the total number of participants receiving that intervention, while the thickness of the connecting lines corresponds to the number of studies enrolled to each direct comparison. The numerical label on each line denotes the number of studies included in that specific comparison.

Three articles involving 8,122 patients investigated the effect of three AODs on the change in eGFR (Figure 4A) (Langdahl et al., 2022; Miller et al., 2007; Saag et al., 2017). Our analysis revealed that the three AODs did not have a significant effect on eGFR compared with placebo (Figure 4B; Supplementary Table S9; sclerostin inhibitors: mean difference 0.50, 95% CI −0.23 to 1.23; PTH analogs: mean difference 0.39, 95% CI −1.31 to 2.09; bisphosphonates: mean difference −0.10, 95% CI −1.13 to 0.93). Treatment efficacy was evaluated on the basis of P-scores. Sclerostin inhibitors exhibited the highest effectiveness, followed by PTH analogs, placebo, and bisphosphonates (Supplementary Table S5).

### 3.6 Risk of bias in included studies

**FIGURE 5:**
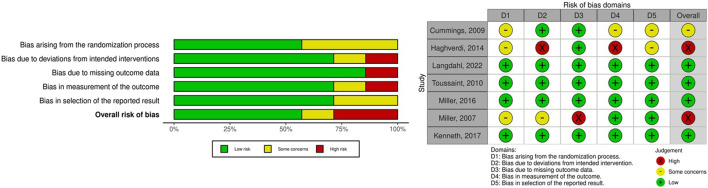
Risk of bias in included studies.

We examined the risk of bias of the seven included studies (Sterne et al., 2019). Two studies were categorized as high risk of bias. The study by Haghverdi et al. (2014) demonstrated a high risk of bias due to deviations from the intended interventions, which people delivering the interventions might be aware of intervention groups during the trial, and bias in the measurement of outcomes, which the measurement or ascertainment of the outcome could have differed between intervention groups. In addition, the study by Miller et al. (2007) exhibited a high risk of bias due to missing outcome data, which the result might be biased by missing outcome data. Four studies were categorized as having a low risk of bias, and one study was classified as having some concerns regarding bias. Figure 5 presents a summary of the risk of bias assessment. Because the number of included studies in each analysis was less than 10, we did not analyze publication bias. The confidence in the current NMA regarding the effectiveness of AODs in preventing clinical fractures compared with placebo was rated from low to very low (Supplementary Table S10). This rating was primarily due to imprecision and heterogeneity. For imprecise evaluation, we referenced a previously published large-scale study in the general population. The study determined that compared with placebo, the risk ratio for clinical fracture effectiveness of AODs ranged from 0.4 to 0.8 (Händel et al., 2023). Accordingly, we set our clinical effectiveness threshold for clinical fracture risk ratio at 0.6. Owing to limited number of enrolled studies and clinical events, the imprecise and heterogeneity domain in most comparisons were mostly some concern or major concern.

## 4 Discussion

This NMA included seven randomized controlled trials involving 18,503 patients, most of whom were postmenopausal women. The analysis revealed that AODs effectively reduced the risk of clinical fractures, with sclerostin inhibitors demonstrating the highest therapeutic efficacy (RR 0.38 with 95% CI 0.23–0.62), following by denosumab (RR 0.58 with 95% CI 0.52–0.66) and bisphosphonates (RR 0.53 with 95% CI 0.30–0.92). Second, three AODs (sclerostin inhibitors, bisphosphonates and PTH analogs) significantly improved BMD at the lumbar spine, total hip, and femoral neck, with PTH analogs exhibiting the highest treatment efficacy. Furthermore, AODs did not significantly affect eGFR. First, our findings demonstrate that by current available, the four AODs significantly reduced the risks of clinical fractures in the CKD population. Compared to placebo, sclerostin inhibitors reduced the clinical fracture rate from 11.8% to 2.8%, with a number needed to treat of 12. In comparison with placebo, the number needed to treat for bisphosphonates group and denosumab group are 16 and 36, respectively. Previous studies have reported that SERMs, bisphosphonates, denosumab, PTH analogs, and sclerostin inhibitors all effectively reduce the risk of fractures, including vertebral, nonvertebral, and hip fractures, in postmenopausal women without CKD (Davis et al., 2020; Author Anonymous, 2021). However, evidence in the CKD population is inconsistent. Hara et al. examined the efficacy of various AODs in patients with CKD stages 3–5 who also had osteoporosis. Their results indicated that different types of pharmacological interventions, including PTH analogs, bisphosphonates, denosumab, and SERMs, reduced the risk of vertebral fractures. However, they observed little or no difference in clinical fracture risk following treatment with AODs (Hara et al., 2021). In our study, we noted that the four types of AODs significantly reduced the risks of clinical fractures in the CKD population. This finding can be attributed to the inclusion of more clinical fracture data from patients with CKD in our study compared with previous studies. Moreover, we noted that sclerostin inhibitors demonstrated the highest treatment efficacy, whereas PTH analogs exhibited relatively low effectiveness in reducing the risk of clinical fractures. Previous studies have reported that PTH analogs and sclerostin inhibitors, which are FDA-approved osteoanabolics, stimulate bone formation to increase bone density, thereby reducing fracture risk (Korff et al., 2023; Li et al., 2021; Lim and Bolster, 2022). The difference in our findings might be attributed to the use of PTH analogs in patients with low bone turnover rates, such as those with adynamic bone disease or mineral and bone disorders, potentially leading to increased bone turnover and higher fracture risk. By contrast, sclerostin inhibitors are less associated with this risk, making them more effective than PTH analogs in reducing clinical fracture risk (Bover et al., 2019; Bover et al., 2014; Giamalis et al., 2015). However, the literature on this topic is limited, and additional research is needed to substantiate these hypotheses.

Second, our analysis revealed that the several AODs significantly improved BMD at the lumbar spine, total hip, and femoral neck, with PTH analogs exhibiting the highest treatment efficacy among the AODs. The ACTIVE study in 2016 demonstrated that abaloparatide increased lumbar spine and total hip BMDs, and teriparatide enhanced lumbar spine BMD in postmenopausal women with osteoporosis (Miller et al., 2016). Consistent with these findings, our analysis demonstrated the superior effect of PTH analogs on BMD improvement. Eastell et al. identified that early changes in serum procollagen type I N propeptide were associated with improvement in lumbar spine BMD in postmenopausal women with osteoporosis receiving PTH analogs (Eastell et al., 2019). This correlation may explain our findings because the majority of the patients in our study were postmenopausal women with CKD (99.5%). Moreover, SERMs resulted in a notable change in mean femoral neck BMD, although we observed no significant difference between the effect of SERMs and those of other AODs. However, this finding should be cautiously interpreted due to the high risk of bias (Figure 5). The MORE study in 1999 (Ettinger et al., 1999) demonstrated that the effect of raloxifene on improvement in femoral neck and lumbar BMDs among postmenopausal women with osteoporosis was associated with lower creatinine clearance (CrCl; CrCl <45 mL/min) (Ishani et al., 2008). The study also reported that raloxifene increased femoral axial and bending strength by 1%–2% and reduced the buckling ratio, an index of cortical instability, by 2% (Uusi-Rasi et al., 2006).

Third, this analysis revealed that three AODs did not significantly affect eGFR. A previous study indicated that an eGFR below 30 mL/min/1.73 m^2^ is considered a contraindication for the use of intravenous bisphosphonates in patients with CKD (Markowitz et al., 2003). In addition, Robinson et al. determined that bisphosphonates increased the risk of CKD progression in adults with moderate to severe CKD (stages 3b–5) (Robinson et al., 2021). However, another study suggested that the use of oral bisphosphonates to treat patients with moderate to severe CKD did not result in adverse renal effects during several years of follow-up (Jamal et al., 2007). The results of this study support the idea that the use of bisphosphonates does not substantially affect CKD stage progression. However, because of the limited number of studies and patients included in this analysis, further evidence from additional studies is required. Regarding PTH analogs, one of the studies included in this analysis indicated that treatment with only teriparatide at 40 µg/day in patients with mild CKD resulted in a significant improvement in eGFR, whereas other interventions had no substantial effect and may lead to adverse events, such as hypercalcemia and hyperuricemia (Miller et al., 2007). Our analysis suggests that PTH analogs may slightly increase eGFR values but do not significantly affect CKD progression. A recent study revealed that romosozumab does not considerably affect eGFR (Miller et al., 2022). However, romosozumab is associated with an increased risk of cardiovascular events so should be used with caution (Cosman et al., 2016). Consistent with the findings of other studies, our analysis indicates that romosozumab may slightly increase eGFR but does not significantly affect CKD progression.

This is the first systematic review and NMA to investigate the differential therapeutic effects of various AODs in patients with CKD and concurrent osteopenia or osteoporosis on clinical fracture risk; mean change in BMD at the lumbar spine, total hip, and femoral neck; and eGFR. Limitations of this analysis include that one of the included studies involved patients undergoing hemodialysis (Haghverdi et al., 2014). Thus, caution is required when interpreting its results. Second, the majority of patients included in this NMA were postmenopausal women, limiting the applicability of our findings to the broader population, including men, children, and premenopausal women. The third limitation is that the analysis did not involve a large number of patients with severe CKD, which leaves some uncertainty regarding the efficacy of AODs in more severe CKD cases. Fourth, most of the included studies compared treatment drugs with placebos, providing limited evidence of the differences between various AODs. Fifth, few studies explored SERMs, with RR values failing to exhibit significant differences in statistical analyses. Finally, this study could not tell the benefits of different AODs among different CKD stages. In Sabaghian et al. study, the meta-analysis reported that AODs could significantly decrease vertebral fracture risk in patients with CKD stage 1–3, but show no apparent benefits in patients with CKD stage 4–5 (Sabaghian et al., 2024). Additionally, Chen et al. revealed that teriparatide and denosumab were most effective in improving BMD of vertebrae and femoral neck, respectively, among patients with CKD including dialysis and kidney transplantation (Chen et al., 2022). We did not conduct a grey literature for unpublished articles. Further NMA study is needed to compare the therapeutic effects of different AODs in patients with different CKD stages. This indicates a need for more comprehensive research to fully understand the effect of AODs in this context. Moreover, analyzing the effects of AODs in the future could be beneficial for specific populations, such as kidney transplant recipients.

## 5 Conclusion

Our NMA examined the differential therapeutic effects of AODs in patients with CKD and concurrent osteopenia or osteoporosis. The studies we examined mainly focused on early CKD stages and included a predominantly female population. The analysis revealed that sclerostin inhibitors might be the most effective pharmacological intervention for preventing clinical fractures in this group. In addition, we observed that AODs do not significantly affect eGFR, neither improving nor worsening renal function. Further studies should examine whether patients with different CKD stages, phenotypes, and mineral and bone disorders benefit from specific AODs. These topics warrant investigation because they can contribute to a more comprehensive body of knowledge and aid in the treatment of patients with CKD.

## Data Availability

The original contributions presented in the study are included in the article/Supplementary Material, further inquiries can be directed to the corresponding authors.
