# Supplementary material for: Effects of anti-osteoporotic drugs in patients with chronic kidney disease: a systemic review and network meta-analysis of bone mineral density, clinical fracture rate and renal function
Source: Front Pharmacol. 2025 Jun 17;16:1569744. doi: 10.3389/fphar.2025.1569744 (PMC12208862; doi:10.3389/fphar.2025.1569744)
Supplement: Supplementary file 1 [file Supplementaryfile1.docx]

***Supplementary Material***

**Supplementary Tables:**

**Supplementary Table 1. PRISMA extension checklist of current network meta-analysis**

**Supplementary Table 2. Search strategy for each database**

**Supplementary Table 3. List of excluded studies**

**Supplementary Table 4. League table of fracture**

**Supplementary Table 5. Clinical P-score of fracture, lumbar spine, total hip, and femoral neck**

**Supplementary Table 6. League table of lumbar spine BMD**

**Supplementary Table 7. League table of total hip BMD**

**Supplementary Table 8. League table of femoral neck BMD**

**Supplementary Table 9. League table of eGFR**

**Supplementary Table 10. Confidence of network meta-analysis**

**Supplementary Figures:**

**Supplementary Figure 1. Lumbar spine BMD network plot**

**Supplementary Figure 2. Total hip BMD network plot**

**Supplementary Figure 3. Femoral neck BMD network plot**

**Supplementary Tables:**

**Supplementary Table 1. PRISMA extension checklist of current network meta-analysis**

| **Section and Topic** | **Item #** | **Checklist item** | **Location where item is reported (section)** |
| --- | --- | --- | --- |
| **TITLE** | | |  |
| Title | 1 | Identify the report as a systematic review. | Title |
| **ABSTRACT** | | |  |
| Abstract | 2 | See the PRISMA 2020 for Abstracts checklist. | Abstract |
| **INTRODUCTION** | | |  |
| Rationale | 3 | Describe the rationale for the review in the context of existing knowledge. | Introduction |
| Objectives | 4 | Provide an explicit statement of the objective(s) or question(s) the review addresses. | Introduction |
| **METHODS** | | |  |
| Eligibility criteria | 5 | Specify the inclusion and exclusion criteria for the review and how studies were grouped for the syntheses. | Study eligibility criteria |
| Information sources | 6 | Specify all databases, registers, websites, organisations, reference lists and other sources searched or consulted to identify studies. Specify the date when each source was last searched or consulted. | The literature search strategy |
| Search strategy | 7 | Present the full search strategies for all databases, registers and websites, including any filters and limits used. | The literature search strategy |
| Selection process | 8 | Specify the methods used to decide whether a study met the inclusion criteria of the review, including how many reviewers screened each record and each report retrieved, whether they worked independently, and if applicable, details of automation tools used in the process. | Study eligibility criteria |
| Data collection process | 9 | Specify the methods used to collect data from reports, including how many reviewers collected data from each report, whether they worked independently, any processes for obtaining or confirming data from study investigators, and if applicable, details of automation tools used in the process. | Data extraction and outcomes |
| Data items | 10a | List and define all outcomes for which data were sought. Specify whether all results that were compatible with each outcome domain in each study were sought (e.g. for all measures, time points, analyses), and if not, the methods used to decide which results to collect. | Data extraction and outcomes |
|  | 10b | List and define all other variables for which data were sought (e.g. participant and intervention characteristics, funding sources). Describe any assumptions made about any missing or unclear information. | Data extraction and outcomes |
| Study risk of bias assessment | 11 | Specify the methods used to assess risk of bias in the included studies, including details of the tool(s) used, how many reviewers assessed each study and whether they worked independently, and if applicable, details of automation tools used in the process. | Data synthesis and statistical analysis |
| Effect measures | 12 | Specify for each outcome the effect measure(s) (e.g. risk ratio, mean difference) used in the synthesis or presentation of results. | Data synthesis and statistical analysis |
| Synthesis methods | 13a | Describe the processes used to decide which studies were eligible for each synthesis (e.g. tabulating the study intervention characteristics and comparing against the planned groups for each synthesis (item #5)). | Figure 1. |
|  | 13b | Describe any methods required to prepare the data for presentation or synthesis, such as handling of missing summary statistics, or data conversions. | Figure 1. |
|  | 13c | Describe any methods used to tabulate or visually display results of individual studies and syntheses. | Table 1. |
|  | 13d | Describe any methods used to synthesize results and provide a rationale for the choice(s). If meta-analysis was performed, describe the model(s), method(s) to identify the presence and extent of statistical heterogeneity, and software package(s) used. | Data synthesis and statistical analysis |
|  | 13e | Describe any methods used to explore possible causes of heterogeneity among study results (e.g. subgroup analysis, meta-regression). | Data synthesis and statistical analysis |
|  | 13f | Describe any sensitivity analyses conducted to assess robustness of the synthesized results. | N/A |
| Reporting bias assessment | 14 | Describe any methods used to assess risk of bias due to missing results in a synthesis (arising from reporting biases). | Data synthesis and statistical analysis |
| Certainty assessment | 15 | Describe any methods used to assess certainty (or confidence) in the body of evidence for an outcome. | Data synthesis and statistical analysis |
| **RESULTS** | | |  |
| Study selection | 16a | Describe the results of the search and selection process, from the number of records identified in the search to the number of studies included in the review, ideally using a flow diagram. | Figure 1. |
|  | 16b | Cite studies that might appear to meet the inclusion criteria, but which were excluded, and explain why they were excluded. | N/A |
| Study characteristics | 17 | Cite each included study and present its characteristics. | Results of the search |
| Risk of bias in studies | 18 | Present assessments of risk of bias for each included study. | Risk of bias in included studies |
| Results of individual studies | 19 | For all outcomes, present, for each study: (a) summary statistics for each group (where appropriate) and (b) an effect estimate and its precision (e.g. confidence/credible interval), ideally using structured tables or plots. | Table 1. |
| Results of syntheses | 20a | For each synthesis, briefly summarise the characteristics and risk of bias among contributing studies. | Results |
|  | 20b | Present results of all statistical syntheses conducted. If meta-analysis was done, present for each the summary estimate and its precision (e.g. confidence/credible interval) and measures of statistical heterogeneity. If comparing groups, describe the direction of the effect. | Results |
|  | 20c | Present results of all investigations of possible causes of heterogeneity among study results. | N/A |
|  | 20d | Present results of all sensitivity analyses conducted to assess the robustness of the synthesized results. | N/A |
| Reporting biases | 21 | Present assessments of risk of bias due to missing results (arising from reporting biases) for each synthesis assessed. | Risk of bias in included studies |
| Certainty of evidence | 22 | Present assessments of certainty (or confidence) in the body of evidence for each outcome assessed. | Supplementary Table 10. |
| **DISCUSSION** | | |  |
| Discussion | 23a | Provide a general interpretation of the results in the context of other evidence. | Discussion |
|  | 23b | Discuss any limitations of the evidence included in the review. | Discussion |
|  | 23c | Discuss any limitations of the review processes used. | N/A |
|  | 23d | Discuss implications of the results for practice, policy, and future research. | Conclusion |
| **OTHER INFORMATION** | | |  |
| Registration and protocol | 24a | Provide registration information for the review, including register name and registration number, or state that the review was not registered. | The Literature search strategy |
|  | 24b | Indicate where the review protocol can be accessed, or state that a protocol was not prepared. | The Literature search strategy |
|  | 24c | Describe and explain any amendments to information provided at registration or in the protocol. | N/A |
| Support | 25 | Describe sources of financial or non-financial support for the review, and the role of the funders or sponsors in the review. | Funding |
| Competing interests | 26 | Declare any competing interests of review authors. | Conflict of Interest |
| Availability of data, code and other materials | 27 | Report which of the following are publicly available and where they can be found: template data collection forms; data extracted from included studies; data used for all analyses; analytic code; any other materials used in the review. | Table 1. |

**Supplementary Table 2. Search strategy for each database**

| PubMed through 5^th^ June, 2023 | | | | |
| --- | --- | --- | --- | --- |
| # | Search term | Filter | | Results |
| 1 | chronic kidney disease |  | |  |
| 2 | (Renal Insufficiency, Chronic)[Mesh] |  | |  |
| 3 | (Renal Dialysis)[Mesh] |  | |  |
| 4 | ESRD OR ESKD |  | |  |
| 5 | (Kidney Transplantation)[Mesh] |  | |  |
| 6 | OR (#1-#5) | - | | 354845 |
| 7 | (osteoporosis)[Mesh] |  | |  |
| 8 | bone mineral density |  | |  |
| 9 | "Chronic Kidney Disease-Mineral and Bone Disorder"[Mesh] |  | |  |
| 10 | OR (#7-#9) | - | | 144927 |
| 11 | clodronate |  | |  |
| 12 | tiludronate |  | |  |
| 13 | alendronate |  | |  |
| 14 | risedronate |  | |  |
| 15 | ibandronate |  | |  |
| 16 | pamidronate |  | |  |
| 17 | zoledronate |  | |  |
| 18 | etidronate |  | |  |
| 19 | diphosphonates[Mesh] |  | |  |
| 20 | bone density conservation agents[Mesh] |  | |  |
| 21 | selective estrogen receptor modulators[Mesh] |  | |  |
| 22 | raloxifene hydrochloride[Mesh] |  | |  |
| 23 | raloxifene |  | |  |
| 24 | bazedoxifene |  | |  |
| 25 | denosumab[Mesh] |  | |  |
| 26 | denosumab OR prolia OR xgeva |  | |  |
| 27 | abaloparatide |  | |  |
| 28 | teriparatide[Mesh] |  | |  |
| 29 | PTH related peptide |  | |  |
| 30 | romosozumab |  | |  |
| 31 | strontium ranelate |  | |  |
| 32 | OR (#11-#31) | - | | 52402 |
| 33 | AND (#6, #10, #32) | Randomized Controlled Trial, Human, Adult age 19+, 1998-2023/06/05, English | | 87 |
| Embase through 5^th^ June, 2023 | | | | |
| # | Search term | Filter | | Results |
| 1 | chronic kidney failure/br |  | |  |
| 2 | chronic kidney disease/br |  | |  |
| 3 | end stage renal disease /br |  | |  |
| 4 | end stage kidney disease /br |  | |  |
| 5 | dialysis/br |  | |  |
| 6 | hemodialysis/br |  | |  |
| 7 | peritoneal dialysis/br |  | |  |
| 8 | kidney transplantation/br |  | |  |
| 9 | renal transplantation/br |  | |  |
| 10 | OR (#1-#9) | - | | 832955 |
| 11 | osteoporosis/exp |  | |  |
| 12 | chronic kidney disease-mineral and bone disorder/exp |  | |  |
| 13 | renal osteodystrophy/ti.ab.kw |  | |  |
| 14 | bone mineral density/exp |  | |  |
| 15 | OR (#11-#14) | - | | 220716 |
| 16 | diphosphonates/exp |  | |  |
| 17 | etidronate OR zoledronate OR pamidronate/ti.ab.kw |  | |  |
| 18 | ibandronate OR risedronate OR alendronate/ti.ab.kw |  | |  |
| 19 | tiludronate OR clodronate/ti.ab.kw |  | |  |
| 20 | raloxifene OR ralozifene hydrochloride/ti.ab.kw |  | |  |
| 21 | selective estrogen receptor modulator/exp |  | |  |
| 22 | selective estrogen receptor degrader/exp |  | |  |
| 23 | selective estrogen receptor downregulator/exp |  | |  |
| 24 | bazedoxifene/ti.ab.kw |  | |  |
| 25 | denosumab/br |  | |  |
| 26 | denosumab/ti.ab.kw |  | |  |
| 27 | xgeva OR prolia/ti.ab.kw |  | |  |
| 28 | teriparatide/br |  | |  |
| 29 | teriparatide/ti.ab.kw |  | |  |
| 30 | abaloparatide/br |  | |  |
| 31 | abaloparatide/ti.ab.kw |  | |  |
| 32 | parathyroid hormone derivative/br |  | |  |
| 33 | romosozumab/br |  | |  |
| 34 | romosozumab/ti.ab.kw |  | |  |
| 35 | strontium ranelate'/br |  | |  |
| 36 | strontium ranelate'/ti.ab.kw |  | |  |
| 37 | OR (#15-#35) | - | | 160823 |
| 38 | AND (#10, #15, #37) | Adult, aged, Randomized Controlled Trial, 1998-2023 | | 156 |
| Web of Science through 5^th^ June, 2023 | | | | |
| # | Query | | Results | |
| 1 | ALL=(chronic kidney disease) | |  | |
| 2 | ALL=(end stage renal disease) | |  | |
| 3 | ALL=(end stage kidney disease) | |  | |
| 4 | ALL=(hemodialysis) | |  | |
| 5 | ALL=(dialysis) | |  | |
| 6 | ALL=(peritoneal dialysis) | |  | |
| 7 | ALL=(renal transplantation) | |  | |
| 8 | ALL=(kidney transplantation) | |  | |
| 9 | OR (#1-#8) | | 427037 | |
| 10 | ALL=(osteoporosis) | |  | |
| 11 | ALL=(bone mineral density) | |  | |
| 12 | ALL=(chronic kidney disease-mineral and bone disorder) | |  | |
| 13 | OR (#10-#12) | | 164025 | |
| 14 | ALL=(diphosphonates OR biphosphonates) | |  | |
| 15 | AB=(etidronate OR zoledronate OR pamidronate) | |  | |
| 16 | AB=(ibandronate OR risedronate OR alendronate) | |  | |
| 17 | AB=(tiludronate OR clodronate) | |  | |
| 18 | ALL=(selective estrogen receptor degrader) | |  | |
| 19 | ALL=(selective estrogen receptor modulator) | |  | |
| 20 | AB=ralozxifene | |  | |
| 21 | AB=bazedoxifene | |  | |
| 22 | ALL=(denosumab) | |  | |
| 23 | TI=(denosumab) | |  | |
| 24 | AB=(denosumab) | |  | |
| 25 | AB=(prolia OR xgeva) | |  | |
| 26 | AB=(teriparatide) | |  | |
| 27 | AB=(abaloparatide) | |  | |
| 28 | ALL=(romosozumab) | |  | |
| 29 | AB=(romosozumab) | |  | |
| 30 | AB=(strontium ranelate) | |  | |
| 31 | OR (#14-#30) | | 33061 | |
| 32 | ALL=(random* AND (control* OR clinical) AND (trial* OR study)) | | 967433 | |
| 33 | AND (#9, #13, #31, #32) | | 101 | |
| 34 | #33 NOT TI=(systemic review)) NOT TI=(meta analysis) | | 86 | |

**Supplementary Table 3. List of excluded studies**

| Number | Reference | Reason for exclusion |
| --- | --- | --- |
| 1 | Aggarwal, H.K., Jain, D., Chhabra, P. & Yadav, R.K. Effects of Short Term Alendronate Administration on Bone Mineral Density in Patients with Chronic Kidney Disease. Pril (Makedon Akad Nauk Umet Odd Med Nauki) 39, 5-13 (2018). | Single arm |
| 2 | Bergner, R. Bisphosphonates in the treatment of renal osteodystrophy. Osteologie 17, 154-158 (2008). | Not RCT |
| 3 | Broadwell, A., et al. Denosumab Safety and Efficacy Among Participants in the FREEDOM Extension Study With Mild to Moderate Chronic Kidney Disease. Journal of Clinical Endocrinology & Metabolism 106, 397-409 (2021). | No outcome of interest |
| 4 | Ishani, A., Blackwell, T., Jamal, S.A., Cummings, S.R. & Ensrud, K.E. The effect of raloxifene treatment in postmenopausal women with CKD. Journal of the American Society of Nephrology : JASN 19, 1430-1438 (2008). | No outcome of interest |
| 5 | Jamal, S.A., et al. Effects of denosumab on fracture and bone mineral density by level of kidney function. Journal of bone and mineral research : the official journal of the American Society for Bone and Mineral Research 26, 1829-1835 (2011). | No outcome of interest |
| 6 | Kikuchi, Y., et al. Effect of risedronate on high-dose corticosteroid-induced bone loss in patients with glomerular disease. Nephrology Dialysis Transplantation 22, 1593-1600 (2007). | Single arm |
| 7 | Martin, K.J., et al. Velcalcetide (AMG 416), a novel peptide agonist of the calcium-sensing receptor, reduces serum parathyroid hormone and FGF23 levels in healthy male subjects. Nephrology, dialysis, transplantation : official publication of the European Dialysis and Transplant Association - European Renal Association 29, 385-392 (2014). | Not target population  (healthy adult male) |
| 8 | Melamed, M.L., et al. Raloxifene, a selective estrogen receptor modulator, is renoprotective: a post-hoc analysis. Kidney Int 79, 241-249 (2011). | Not target population (post-menopausal women with osteoporosis) |
| 9 | Miller, D., et al. Efficacy and safety of romosozumab among postmenopausal women with osteoporosis and mild-to-moderate chronic kidney disease. Osteoporosis International 31, S41 (2020). | Duplication |
| 10 | Shigematsu, T., Muraoka, R., Sugimoto, T. & Nishizawa, Y. Risedronate therapy in patients with mild-to-moderate chronic kidney disease with osteoporosis: post-hoc analysis of data from the risedronate phase III clinical trials. Bmc Nephrology 18(2017). | Single arm |
| 11 | Sugimoto, T., et al. Efficacy and safety of once-monthly risedronate in osteoporosis subjects with mild-to-moderate chronic kidney disease: a post hoc subgroup analysis of a phase III trial in Japan. Journal of Bone and Mineral Metabolism 37, 730-740 (2019). | Single arm |
| 12 | Bilezikian, J.P., Fitzpatrick, L.A., Williams, G.C., Hu, M.Y., Hattersley, G. & Rizzoli, R.. Bone mineral density and bone turnover marker changes with sequential abaloparatide/alendronate: Results of ACTIVExtend [abstract no: MO0655]. Journal of Bone & Mineral Research 32(1):S377 (2017). | Duplication |
| 13 | Bilezikian, J.P., Hattersley, G., Williams, G., Hu, M.Y., Fitzpatrick, L.A. & Papapoulos, S.. Abaloparatide-SC has minimal effects in subjects with mild or moderate renal impairment: Results from the ACTIVE trial [abstract no: SU0282]. Journal of Bone & Mineral Research 31(Suppl 1):S264 (2016). | Duplication |
| 14 | Cosman, F., Hattersley, G., Hu, M.Y., Williams, G.C., Fitzpatrick, L.A., Black, D.M.. Effects of abaloparatide-sc on fractures and bone mineral density in subgroups of postmenopausal women with osteoporosis and varying baseline risk factors. Journal of Bone & Mineral Research 32(1):17-23 (2017). | Duplication |
| 15 | Moreira, C.A., Fitzpatrick, L.A., Wang, Y., Recker, R.R. Effects of abaloparatide-SC (BA058) on bone histology and histomorphometry: the ACTIVE phase 3 trial. Bone 97:314-9 (2017). | Duplication |
| 16 | Black, D.M., Cummings, S.R., Karpf, D.B., Cauley, J.A., Thompson, D.E.& Nevitt, M.C., et al. Randomised trial of effect of alendronate on risk of fracture in women with existing vertebral fractures. Fracture Intervention Trial Research Group. Lancet 348(9041):1535-41 (1996). | Not target population  (Women aged 55-81 with low femoral-neck BMD) |
| 17 | Black, D.M., Reiss, T.F., Nevitt, M.C., Cauley, J., Karpf, D., Cummings, S.R.. Design of the Fracture Intervention Trial. Osteoporosis International 3 Suppl 3:S29-39 (1993). | Not target population  (Women aged 55-81 with low femoral-neck BMD) |
| 18 | Cummings, S.R., Black, D.M., Thompson, D.E., Applegate, W.B., Barrett-Connor, E., Musliner, T.A., et al. Effect of alendronate on risk of fracture in women with low bone density but without vertebral fractures: results from the Fracture Intervention Trial. JAMA 280(24):2077-82 (1998). | Not target population  (Women aged 55-81 with low femoral-neck BMD) |
| 19 | Ensrud, K.E., Barrett-Connor, E.L., Schwartz, A., Santora, A.C., Bauer, D.C., Suryawanshi, S., et al. Randomized trial of effect of alendronate continuation versus discontinuation in women with low BMD: results from the Fracture Intervention Trial long-term extension. Journal of Bone & Mineral Research 19(8):1259-69 (2004). | Not target population  (Women aged 55-81 with low femoral-neck BMD) |
| 20 | Jamal, S.A., Bauer, D.C., Ensrud, K.E., Cauley, J.A., Hochberg, M., Ishani, A., et al. Alendronate treatment in women with normal to severely impaired renal function: an analysis of the fracture intervention trial. Journal of Bone & Mineral Research 22(4):503-8 (2007). | Single arm |
| 21 | Egbuna, O.I., Cheung, A.M., Siddhanti, S., Wang, A., Daizadeh, N., Anthony, M., et al. Treatment of osteoporosis by RANKL inhibition with denosumab in women at high cardiovascular risk and with renal impairment does not accelerate vascular calcification  [abstract no: SA-PO2319]. Journal of the American Society of Nephrology 21(Abstract Suppl):640A (2010). | No outcome of interest |
| 22 | Boonen, S., Marin, F., Mellstrom, D., Xie, L., Desaiah, D., Krege, J.H., et al. Safety and efficacy of teriparatide in elderly women with established osteoporosis: bone anabolic therapy from a geriatric perspective. Journal of the American Geriatrics Society 54(5):782-9 (2006). | Not target population  (elderly women with osteoporosis) |
| 23 | Chen, P., Miller, P.D., Delmas, P.D., Misurski, D.A., Krege, J.H.. Change in lumbar spine BMD and vertebral fracture risk reduction in teriparatide-treated postmenopausal women with osteoporosis. Journal of Bone & Mineral Research 21(11):1785-90 (2006). | Not target population  (postmenopausal women with osteoporosis) |
| 24 | Crans, G.G., Silverman, S.L., Genant, H.K., Glass, E.V., Krege, J.H.. Association of severe vertebral fractures with reduced quality of life: reduction in the incidence of severe vertebral fractures by teriparatide. Arthritis & Rheumatism 50(12):4028-34 (2004). | Not target population  (postmenopausal women with osteoporosis) |
| 25 | Dawson-Hughes, B., Chen, P., Krege, J.H.. Response to teriparatide in patients with baseline 25-hydroxyvitamin D insufficiency or sufficiency. Journal of Clinical Endocrinology & Metabolism 92(12):4630-6 (2007). | Not target population  (postmenopausal women with osteoporosis) |
| 26 | Delmas, P.D., Licata, A.A., Reginster, J.Y., Crans, G.G., Chen, P., Misurski, D.A., et al. Fracture risk reduction during treatment with teriparatide is independent of pretreatment bone turnover. Bone 39(2):237-43 (2006). | Not target population  (postmenopausal women with osteoporosis) |
| 27 | Gallagher, J.C., Genant, H.K., Crans, G.G., Vargas, S.J., Krege, J.H.. Teriparatide reduces the fracture risk associated with increasing number and severity of osteoporotic fractures. Journal of Clinical Endocrinology & Metabolism ;90(3):1583-7 (2005). | Not target population  (postmenopausal women with osteoporosis) |
| 28 | Genant, H.K., Halse, J., Briney, W.G., Xie, L., Glass, E.V., Krege, J.H.. The effects of teriparatide on the incidence of back pain in postmenopausal women with osteoporosis. Current Medical Research & Opinion 21(7):1027-34 (2005). | Not target population  (postmenopausal women with osteoporosis) |
| 29 | Genant, H.K., Siris, E., Crans, G.G., Desaiah, D., Krege, J.H.. Reduction in vertebral fracture risk in teriparatide-treated postmenopausal women as assessed by spinal deformity index. Bone 37(2):170-4 (2005). | Not target population  (postmenopausal women with osteoporosis) |
| 30 | Jiang, Y., Zhao, J.J., Mitlak, B.H., Wang, O., Genant, H.K., Eriksen, E.F.. Recombinant human parathyroid hormone (1-34) [teriparatide] improves both cortical and cancellous bone structure. Journal of Bone & Mineral Research 18(11):1932-41 (2003). | Not target population  (postmenopausal women with osteoporosis) |
| 31 | Krege, J.H., Wan, X.. Teriparatide and the risk of nonvertebral fractures in women with postmenopausal osteoporosis. Bone 50(1):161-4 (2012). | Not target population  (postmenopausal women with osteoporosis) |
| 32 | Neer, R.M., Arnaud, C.D., Zanchetta, J.R., Prince, R., Gaich, G.A., Reginster, J.Y., et al. Effect of parathyroid hormone (1-34) on fractures and bone mineral density in postmenopausal women with osteoporosis. New England Journal Medicine 344(19):1434-41 (2001). | Not target population  (postmenopausal women with osteoporosis) |
| 33 | Paschalis, E.P., Glass, E.V., Donley, D.W., Eriksen, E.F.. Bone mineral and collagen quality in iliac crest biopsies of patients given teriparatide: new results from the Fracture Prevention Trial. Journal of Clinical Endocrinology & Metabolism 90(8):4644-9 (2005). | Not target population  (postmenopausal women with osteoporosis) |
| 34 | Satterwhite, J., Heathman, M., Miller, P.D., Marin, F., Glass, E.V., Dobnig, H.. Pharmacokinetics of teriparatide (rhPTH[1-34]) and calcium pharmacodynamics in postmenopausal women with osteoporosis. Calcified Tissue International87(6):485-92 (2010). | Not target population  (postmenopausal women with osteoporosis) |
| 35 | Silverman, S.L., Piziak, V.K., Chen, P., Misurski, D.A., Wagman, R.B.. Relationship of health related quality of life to prevalent and new or worsening back pain in postmenopausal women with osteoporosis. Journal of Rheumatology 32(12):2405-9 (2005). | Not target population  (postmenopausal women with osteoporosis) |
| 36 | Uusi-Rasi, K., Semanick, L.M., Zanchetta, J.R., Bogado, C.E., Eriksen, E.F., Sato, M., et al. Effects of teriparatide [rhPTH (1-34)] treatment on structural geometry of the proximal femur in elderly osteoporotic women. Bone 36(6):948-58 (2005). | Not target population  (postmenopausal women with osteoporosis) |
| 37 | Watts, N.B., Miller, P.D., Kohlmeier, L.A., Sebba, A., Chen, P., Wong, M., et al. Vertebral fracture risk is reduced in women who lose femoral neck BMD with teriparatide treatment. Journal of Bone & Mineral Research 24(6):1125-31 (2009). | Not target population  (postmenopausal women with osteoporosis) |
| 38 | Weisinger, J.R., Heilberg, I.P., Hernandez, E., Carlini, R., Bellorin-Font, E.. Selective estrogen receptor modulators in chronic renal failure. Kidney International – Supplement 63(85):S62-5 (2003). | Duplication |
| 39 | Borgstrom, F., Johnell, O., Kanis, J.A., Oden, A., Sykes, D., Jonsson, B.. Cost effectiveness of raloxifene in the treatment of osteoporosis in Sweden: an economic evaluation based on the MORE study.  Pharmacoeconomics 22(17):1153-65 (2004;). | Not target population  (postmenopausal women with osteoporosis) |
| 40 | Ettinger, B., Black, D.M., Mitlak, B.H., Knickerbocker, R.K., Nickelsen, T., Genant, H.K., et al. Reduction of vertebral fracture risk in postmenopausal women with osteoporosis treated with raloxifene: results from a 3-year randomized clinical trial. Multiple Outcomes of Raloxifene Evaluation (MORE) Investigators [Erratum in: JAMA 1999 Dec 8;282(22):2124]. JAMA 282(7):637-45 (1999). | Not target population  (postmenopausal women with osteoporosis) |
| 41 | Johnell, O., Kanis, J.A., Black, D.M., Balogh, A., Poor, G., Sarkar, S., et al. Associations between baseline risk factors and vertebral fracture risk in the Multiple Outcomes of Raloxifene Evaluation (MORE) Study. Journal of Bone & Mineral Research 19(5):764-72 (2004). | No outcome of interest |
| 42 | Kanis, J.A., Borgstrom, F., Johnell, O., Oden, A., Sykes, D., Jonsson, B.. Cost-effectiveness of raloxifene in the UK: an economic evaluation based on the MORE study. Osteoporosis International 16(1):15-25 (2005). | Not target population  (postmenopausal women with osteoporosis) |
| 43 | Silverman, S.L., Shen, W., Minshall, M.E., Xie, S., Moses, K.H.. Prevalence of depressive symptoms in postmenopausal women with low bone mineral density and/or prevalent vertebral fracture: results from the Multiple Outcomes of Raloxifene Evaluation (MORE) study. Journal of Rheumatology 34(1):140-4 (2007). | Not target population  (postmenopausal women with osteoporosis) |
| 44 | Siris, E., Adachi, J.D., Lu, Y., Fuerst, T., Crans, G.G., Wong, M., et al. Effects of raloxifene on fracture severity in postmenopausal women with osteoporosis: results from the MORE study. Multiple Outcomes of Raloxifene Evaluation. Osteoporosis International 13(11):907-13 (2002). | Not target population  (postmenopausal women with osteoporosis) |
| 45 | Uusi-Rasi, K., Beck, T.J., Semanick, L.M., Daphtary, M.M., Crans, G.G., Desaiah, D., et al. Structural effects of raloxifene on the proximal femur: results from the Multiple Outcomes of Raloxifene Evaluation trial. Osteoporosis International 17(4):575-86 (2006). | Not target population  (postmenopausal women with osteoporosis) |

**Supplementary Table 4. League table of fracture**

| **Sclerostin inhibitor** | 0.72  (0.54 to 0.95) | - | - | - | 0.39  (0.24 to 0.64) |
| --- | --- | --- | --- | --- | --- |
| 0.72  (0.55 to 0.96) | **Bisphosphonates** | - | - | - | 0.25  (0.01 to 5.27) |
| 0.65  (0.39 to 1.08) | 0.90  (0.51 to 1.60) | **Denosumab** | - | - | 0.58  (0.52 to 0.66) |
| 0.76  (0.03 to 22.64) | 1.05  (0.04 to 31.65) | 1.17  (0.04 to 33.59) | **SERM** | - | 0.50  (0.02 to 14.35) |
| 0.56  (0.32 to 0.96) | 0.77  (0.42 to 1.41) | 0.85  (0.66 to 1.11) | 0.73  (0.03 to 21.13) | **PTH analog** | 0.68  (0.55 to 0.86) |
| 0.38  (0.23 to 0.62) | 0.53  (0.30 to 0.92) | 0.58  (0.52 to 0.66) | 0.50  (0.02 to 14.35) | 0.68  (0.55 to 0.86) | **Placebo** |

PTH: parathyroid hormone. SERM: selective estrogen receptor modulators. Outcome are provided as RR (relative risk) and 95% CI. RRs lower than 1 favor the column-defining intervention for the network meta-analysis results (lower triangle) and the row-defining intervention for the pairwise meta-analysis results (upper triangle)

**Supplementary Table 5. Clinical P-score of fracture, lumbar spine, total hip, and femoral neck**

|  | **Fracture** | **Lumbar spine** | **Total hip** | **Femoral neck** |
| --- | --- | --- | --- | --- |
| **Sclerostin inhibitor** | 0.90 | 0.65 | 0.67 | 0.68 |
| **Bisphosphonates** | 0.59 | 0.31 | 0.33 | 0.32 |
| **Denosumab** | 0.55 | - | - | - |
| **SERM** | 0.54 | 0.52 | - | 0.64 |
| **PTH analog** | 0.35 | 0.97 | 1.00 | 0.80 |
| **Placebo** | 0.07 | 0.05 | 0.00 | 0.06 |

PTH: parathyroid hormone. SERM: selective estrogen receptor modulators.

**Supplementary Table 6. League table of lumbar spine BMD**

| **PTH analog** | - | - | - | 0.07  ( 0.07 to 0.08) |
| --- | --- | --- | --- | --- |
| 0.03  ( 0.03 to 0.04) | **Sclerostin inhibitor** | - | 0.03  ( 0.03 to 0.03) | 0.04  ( 0.04 to 0.04) |
| 0.04  (-0.03 to 0.11) | 0.01  (-0.06 to 0.07) | **SERM** | - | 0.03  (-0.04 to 0.10) |
| 0.06  ( 0.06 to 0.07) | 0.03  ( 0.03 to 0.03) | 0.02  (-0.04 to 0.09) | **Bisphosphonates** | - |
| 0.07  ( 0.07 to 0.08) | 0.04  ( 0.04 to 0.04) | 0.03  (-0.04 to 0.10) | 0.01  ( 0.01 to 0.01) | **Placebo** |

PTH: parathyroid hormone. SERM: selective estrogen receptor modulators. Outcome are provided as RR (relative risk) and 95% CI. MD larger than 0 favor the column-defining intervention (indicates improvement of BMD) for the network meta-analysis results (lower triangle) and the row-defining intervention for the pairwise meta-analysis results (upper triangle).

**Supplementary Table 7. League table of total hip BMD**

| **PTH analog** | - | - | 0.021  (0.019 to 0.024) |
| --- | --- | --- | --- |
| 0.006  (0.003 to 0.009) | **Sclerostin inhibitor** | 0.012  (0.012 to 0.012) | 0.015  (0.015 to 0.016) |
| 0.018  (0.015 to 0.021) | 0.012  (0.012 to 0.012) | **Bisphosphonates** | - |
| 0.021  (0.019 to 0.024) | 0.015  (0.015 to 0.016) | 0.003  (0.003 to 0.004) | **Placebo** |

PTH: parathyroid hormone. SERM: selective estrogen receptor modulators. Outcome are provided as RR (relative risk) and 95% CI. MD larger than 0 favor the column-defining intervention (indicates improvement of BMD) for the network meta-analysis results (lower triangle) and the row-defining intervention for the pairwise meta-analysis results (upper triangle).

**Supplementary Table 8. League table of femoral neck BMD**

| **PTH analog** | - | - | 0.021  (0.019 to 0.024) |
| --- | --- | --- | --- |
| 0.006  (0.003 to 0.009) | **Sclerostin inhibitor** | 0.012  (0.012 to 0.012) | 0.015  (0.015 to 0.016) |
| 0.018  (0.015 to 0.021) | 0.012  (0.012 to 0.012) | **Bisphosphonates** | - |
| 0.021  (0.019 to 0.024) | 0.015  (0.015 to 0.016) | 0.003  (0.003 to 0.004) | **Placebo** |

PTH: parathyroid hormone. SERM: selective estrogen receptor modulators. Outcome are provided as RR (relative risk) and 95% CI. MD larger than 0 favor the column-defining intervention (indicates improvement of BMD) for the network meta-analysis results (lower triangle) and the row-defining intervention for the pairwise meta-analysis results (upper triangle).

**Supplementary Table 9. League table of eGFR**

| **Sclerostin inhibitor** | - | 0.50  (-0.23 to 1.23) | 0.60  (-0.13 to 1.33) |
| --- | --- | --- | --- |
| 0.11  (-1.74 to 1.96) | **PTH analog** | 0.39  (-1.31 to 2.09) | - |
| 0.50  (-0.23 to 1.23) | 0.39  (-1.31 to 2.09) | **Placebo** | - |
| 0.60  (-0.13 to 1.33) | 0.49  (-1.50 to 2.47) | 0.10  (-0.93 to 1.13) | **Bisphosphonates** |

PTH: parathyroid hormone. SERM: selective estrogen receptor modulators. Outcome are provided as RR (relative risk) and 95% CI. MD larger than 0 favor the column-defining intervention (indicates improvement of eGFR) for the network meta-analysis results (lower triangle) and the row-defining intervention for the pairwise meta-analysis results (upper triangle).

**Supplementary Table 10. Confidence of network meta-analysis**

| **Comparison** | **Within-study bias** | **Reporting bias** | **Indirectness** | **Imprecision** | **Heterogeneity** | **Incoherence** | **Confidence rating** |
| --- | --- | --- | --- | --- | --- | --- | --- |
| **Bisphosphonates vs. Placebo** | No concerns | Low risk | No concerns | No concerns | Major concerns | No concerns | Low |
| **Bisphosphonates vs. Sclerostin inhibitor** | No concerns | Low risk | No concerns | No concerns | Major concerns | No concerns | Low |
| **Denosumab vs. Placebo** | Some concerns | Low risk | No concerns | No concerns | Some concerns | No concerns | Low |
| **Placebo vs. PTH analog** | Major concerns | Low risk | No concerns | No concerns | Major concerns | No concerns | Very low |
| **Placebo vs. SERM** | Major concerns | Low risk | No concerns | Major concerns | No concerns | No concerns | Very low |
| **Placebo vs. Sclerostin inhibitor** | No concerns | Low risk | No concerns | No concerns | Major concerns | No concerns | Low |
| **Bisphosphonates vs. Denosumab** | No concerns | Low risk | No concerns | Some concerns | Some concerns | No concerns | Moderate |
| **Bisphosphonates vs. PTH analog** | No concerns | Low risk | No concerns | Some concerns | Some concerns | No concerns | Moderate |
| **Bisphosphonates vs. SERM** | No concerns | Low risk | No concerns | Major concerns | No concerns | No concerns | Low |
| **Denosumab vs. PTH analog** | Some concerns | Low risk | No concerns | No concerns | Major concerns | No concerns | Very low |
| **Denosumab vs. SERM** | Some concerns | Low risk | No concerns | Major concerns | No concerns | No concerns | Very low |
| **Denosumab vs. Sclerostin inhibitor** | No concerns | Low risk | No concerns | Some concerns | Some concerns | No concerns | Moderate |
| **PTH analog vs. SERM** | Major concerns | Low risk | No concerns | Major concerns | No concerns | No concerns | Very low |
| **PTH analog vs. Sclerostin inhibitor** | No concerns | Low risk | No concerns | No concerns | Major concerns | No concerns | Low |
| **Sclerostin inhibitor vs. SERM** | No concerns | Low risk | No concerns | Major concerns | No concerns | No concerns | Low |

**Supplementary Figures:**

**Supplementary Figure 1. Lumbar spine BMD network plot**

**
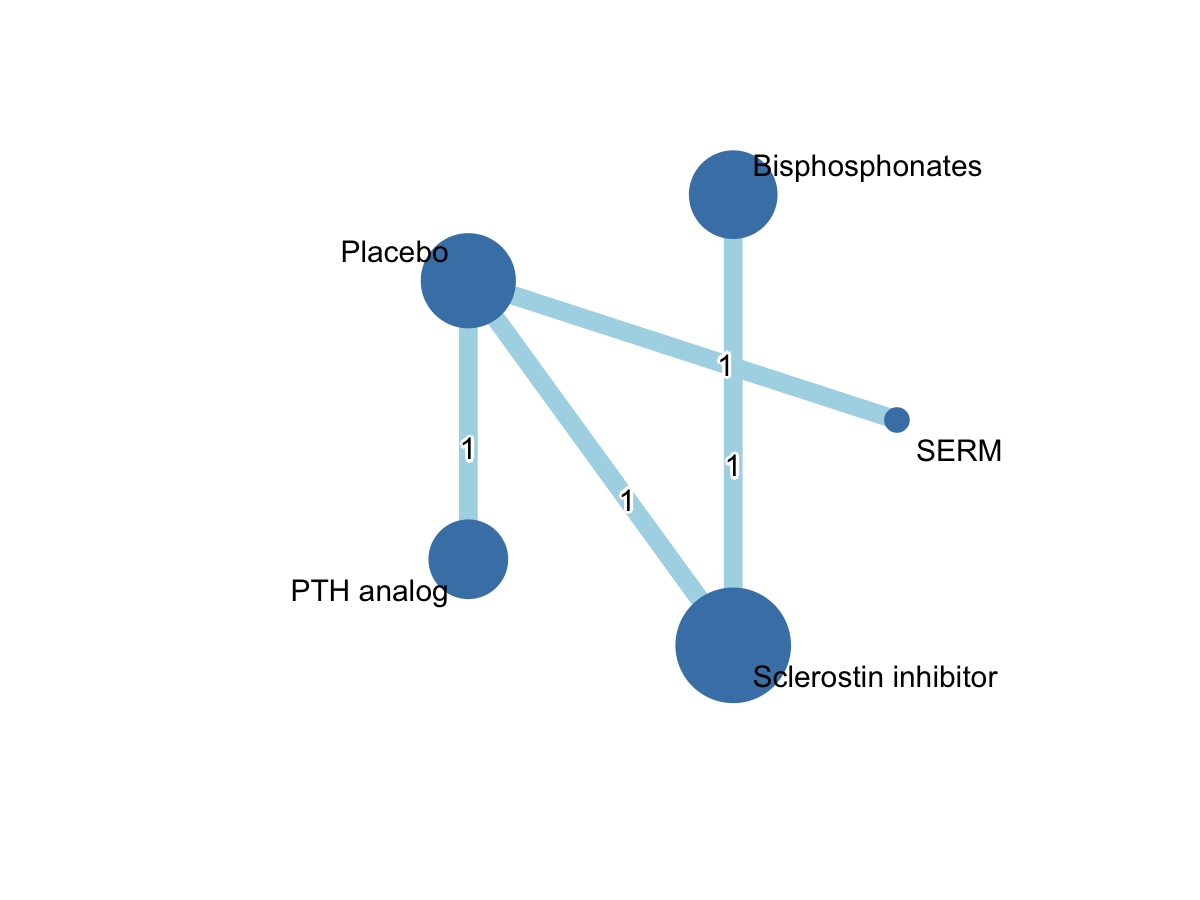
**

PTH: parathyroid hormone. SERM: selective estrogen receptor modulators.

**Supplementary Figure 2. Total hip BMD network plot**

**
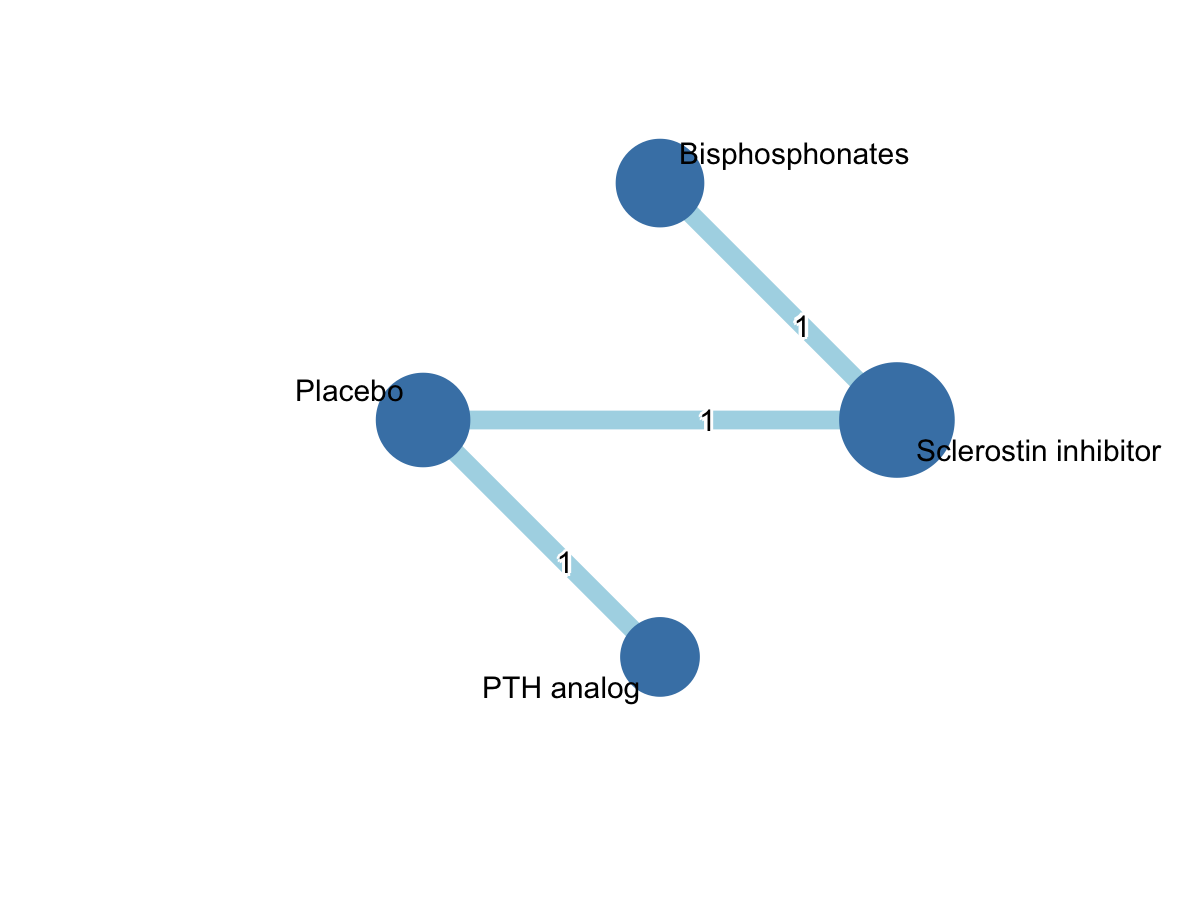
**

PTH: parathyroid hormone.

**Supplementary Figure 3. Femoral neck BMD network plot**


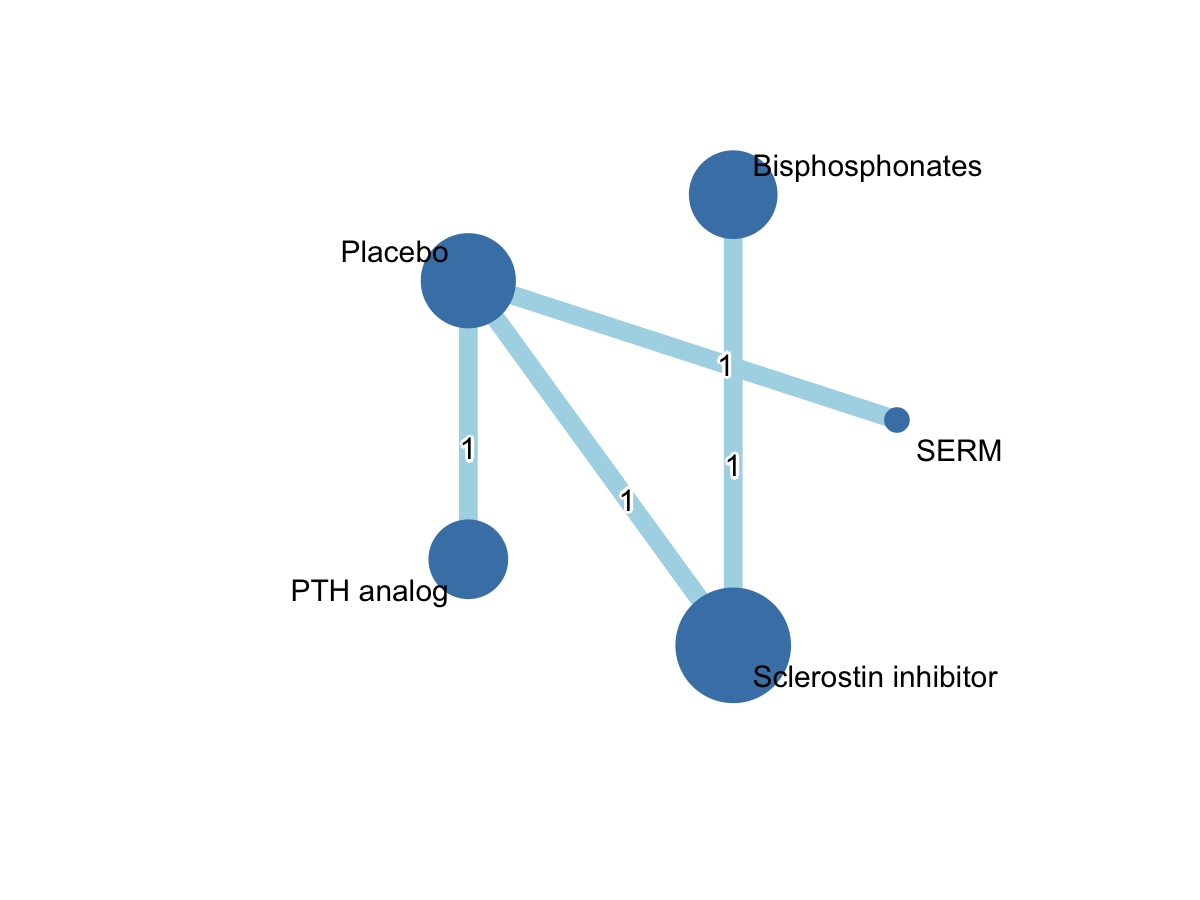


PTH: parathyroid hormone. SERM: selective estrogen receptor modulators.
